# In-hospital Cardiac Arrest in Patients With Sepsis: A National Cohort Study

**DOI:** 10.3389/fmed.2021.731266

**Published:** 2021-10-15

**Authors:** Catherine Duazo, Jo-Ching Hsiung, Frank Qian, Charles Fox Sherrod, Dean-An Ling, I-Ju Wu, Wan-Ting Hsu, Ye Liu, Chen Wei, Babak Tehrani, Tzu-Chun Hsu, Chien-Chang Lee

**Affiliations:** ^1^Department of Medicine, Warren Alpert Medical School of Brown University, Providence, RI, United States; ^2^Department of Medicine, National Taiwan University, Taipei, Taiwan; ^3^Department of Medicine, Beth Israel Deaconess Medical Center, Harvard Medical School, Boston, MA, United States; ^4^Department of Emergency Medicine, National Taiwan University Hospital, Taipei, Taiwan; ^5^Department of Epidemiology, Harvard T.H. Chan School of Public Health, Boston, MA, United States; ^6^Department of Health Care Organization and Policy, University of Alabama at Birmingham, School of Public Health, Birmingham, AL, United States; ^7^Department of Medicine, Harvard Medical School, Boston, MA, United States; ^8^Department of Internal Medicine, Stanford Health Care, Stanford, CA, United States; ^9^Center of Intelligent Healthcare, National Taiwan University Hospital, Taipei, Taiwan; ^10^Byers Center for Biodesign, Stanford University, Stanford, CA, United States

**Keywords:** sepsis, cardiac arrest, revised cardiac index, mortality, in-hospital cardiac arrest

## Abstract

**Background:** Little is known about the risk of in-hospital cardiac arrest (IHCA) among patients with sepsis. We aimed to characterize the incidence and outcome of IHCA among patients with sepsis in a national database. We then determined the major risk factors associated with IHCA among sepsis patients.

**Methods:** We used data from a population-based cohort study based on the National Health Insurance Research Database of Taiwan (NHRID) between 2000 and 2013. We used Martin's implementation that combined the explicit ICD-9 CM codes for sepsis and six major organ dysfunction categories. IHCA among sepsis patients was identified by the presence of cardiopulmonary resuscitation procedures. The survival impact was analyzed with the Cox proportional-hazards model using inverse probability of treatment weighting (IPTW). The risk factors were identified by logistic regression models with 10-fold cross-validation, adjusting for competing risks.

**Results:** We identified a total of 20,022 patients with sepsis, among whom 2,168 developed in-hospital cardiac arrest. Sepsis patients with a higher burden of comorbidities and organ dysfunction were more likely to develop in-hospital cardiac arrest. Acute respiratory failure, hematological dysfunction, renal dysfunction, and hepatic dysfunction were associated with increased risk of IHCA. Regarding the source of infection, patients with respiratory tract infections were at the highest risk, whereas patients with urinary tract infections and primary bacteremia were less likely to develop IHCA. The risk of IHCA correlated well with age and revised cardiac risk index (RCRI). The final competing risk model concluded that acute respiratory failure, male gender, and diabetes are the three strongest predictors for IHCA. The effect of IHCA on survival can last 1 year after hospital discharge, with an IPTW-weighted hazard ratio of 5.19 (95% CI: 5.06, 5.35) compared to patients who did not develop IHCA.

**Conclusion:** IHCA in sepsis patients had a negative effect on both short- and long-term survival. The risk of IHCA among hospitalized sepsis patients was strongly correlated with age and cardiac risk index. The three identified risk factors can help clinicians to identify patients at higher risk for IHCA.

## Introduction

In-hospital cardiac arrest (IHCA) poses major clinical challenges and is a public health burden. Each year, over 290,000 hospitalized patients develop IHCA in the United States (1). A recent analysis of the 2000–2009 American Heart Association (AHA) Get with the Guidelines (GWTG)–Resuscitation registry, showed a median incidence of 4.02 IHCAs per 1,000 hospital admissions [interquartile range (IQR), 2.95–5.65 per 1,000 admissions]. The median survival rate to hospital discharge for this period was 18.8% (2). Internationally, the incidence of IHCA ranged from 1 to 5 per 1,000 hospital admissions with varying survival rates (0–42%) (1). In 2012, a report by National Confidential Enquiry into Patient Outcome and Death (NCEPOD) found that cardiac arrests were predictable in 64%, and potentially avoidable in 38%, of cases (3).

Among the factors that were investigated, pre-arrest sepsis was listed as a risk factor for IHCA. Various studies have reported that the prevalence of sepsis among adult patients with IHCA ranged from 13 to 27% (4, 5). Patients with sepsis are a more vulnerable group compared to the general patient population. Previous studies have found that a high proportion of patients with IHCA had sepsis and multi-organ dysfunction, and their prognosis was worse than the non-sepsis IHCA in general (6). A single-center study in Finland showed that the patients with severe sepsis before IHCA had both higher 30-day mortalities and higher 1-year mortalities than those without sepsis before IHCA (6). However, the heterogeneous patient population, different study settings, and variable sepsis definitions used in each study prevent clear conclusions.

Much less is known regarding the predictors, incidence, and outcome of IHCA among hospitalized sepsis patients. Thus, the aim of this study was 3-fold. First, we aimed to determine the incidence and major risk factors for IHCA among patients hospitalized with sepsis. Next, we aimed to determine the short-term and long-term outcomes for sepsis patients who developed IHCA as compared to patients who did not develop IHCA. Understanding the risk factors for IHCA and quantifying its adverse impact among sepsis patients has important implications to identify opportunities for focusing future efforts on quality improvement including pharmacologic prevention and systems-based practices.

## Methods

### Study Population

We used data from a cohort study constructed from the National Health Insurance Research Database (NHIRD) in Taiwan. Taiwan's National Health Insurance program is a compulsory government-operated health insurance system that covers 99.6% of Taiwanese residents. One million subjects in the NHIRD were randomly selected from the 23 million Taiwanese residents that represent the demographic and geographic distribution of the Taiwanese population. Subjects were longitudinally followed from 2000 to 2013. Patient consent was not required for this study as this was an electronic database study involving anonymous subjects. Ethics approval was granted by the Institutional Review Board of the National Taiwan University Hospital.

### Identification of Sepsis Patients

Sepsis hospitalizations were identified using a validated approach that selected admissions with relevant International Classification of Diseases, Ninth Revision, Clinical Modification (ICD-9-CM) diagnosis/procedure codes. In accordance with the Sepsis-3 definition, sepsis was defined as a life-threatening organ dysfunction caused by a dysregulated host response to infection (7). The coding system proposed and validated previously by Martin et al. (8) was a more conservative estimate that showed a parallel trend with the electronic health record (EHR) estimates (9). Therefore, we used Martin's implementation to identify patients with sepsis in this study. Operationally, we identified cases with sepsis by selecting all cases with explicit ICD-9-CM codes for sepsis or systemic fungal infection (e.g., 038 septicemias, 020.0 septicemic, 790.7 bacteremias, 117.9 disseminated fungal infections, 112.5 disseminated candida infection, or 112.81 disseminated fungal endocarditis) and a diagnosis of acute organ dysfunction. Source of infection was categorized as lower respiratory tract infection, genitourinary tract infection, skin and skin structure infection, catheter-related bloodstream infection, intra-abdominal infection, systemic fungal infection, primary bacteremia, musculoskeletal infection, and biliary tract infection. Acute organ/system dysfunction was categorized as follows: cardiovascular, respiratory, central nervous system (CNS), hematologic, hepatic, renal, and metabolic system dysfunction. The shock was included as a form of cardiovascular dysfunction. The following information was collected for analysis: demographics, presence of pre-existing comorbidity, and outcome.

### Identification of IHCA

We identified IHCA by the presence of the cardiopulmonary resuscitation procedure code.

### Outcome Measurements

The primary outcome of interest was the incidence of sepsis patients complicated by IHCA, stratified by age and Revised Cardiac Risk Index (RCRI) (10). The RCRI was developed to predict the risk of cardiac complications pre-operatively. This index can identify patients at higher risk for complications such as myocardial infarction, pulmonary edema, ventricular fibrillation or primary cardiac arrest, and complete heart block. The RCRI incorporates six independent variables, which include history of ischemic heart disease, heart failure, cerebrovascular disease, diabetes mellitus, chronic kidney disease, and major operations (suprainguinal vascular, intrathoracic, and intraperitoneal). We sought to examine whether RCRI could be used to risk-stratify hospitalized patients for the likelihood of developing IHCA. The secondary outcome was the impact of IHCA on short- and long-term mortality and risk factors for IHCA among sepsis patients. To provide a control for the baseline difference between patients with and without IHCA, we constructed a propensity score (PS) that included patient demographics (such as age, sex, insurance premium, and area of residence), chronic disease conditions, additional clinically relevant baseline patient characteristics (such as alcohol/drug use, psychiatric disorder, neurologic disorder, obesity, bed-ridden status, solid organ transplantation, and atrial fibrillation), sepsis-associated acute organ dysfunctions (such as renal dysfunction, cardiovascular dysfunction/shock, hematologic system dysfunction, acute respiratory failure, CNS dysfunction, and hepatic dysfunction), and various sources of infections (such as lower respiratory tract, genitourinary tract, skin and skin structure, catheter-associated, intra-abdominal, biliary tract, and musculoskeletal).

### Statistical Analysis

Continuous variables that are normally distributed were presented as mean with standard error (SE), and non-normal variables were reported as median with IQR. Categorical variables were reported as a percentage (%). We plotted bar charts to show the risk of IHCA associated with age and RCRI. The correlation between IHCA risk and age categories or RCRI was evaluated by Spearman–rank correlation test. To evaluate the impact of IHCA on the outcome of sepsis patients after discharge, we used the PS weighting method to adjust for differences in baseline comorbidities and sepsis severity. We did not use the PS matching method because there was a high imbalance between the number of patients who developed IHCA and those who did not. PS was defined as the conditional probability of developing IHCA for the index episode of sepsis. The PS was created from a binary logistic regression model that included 29 covariates associated with IHCA. The matching weights (11) were calculated as:


$$\begin{array}{l} Matching\;weight\;\left(MW\right)=\frac{min\;\left(e_{ki}\right)}{\overset{K}{\underset{k=1}{\sum }}I\left(Z_{i}=K\right)e_{ki}} \end{array}$$

where e_ki_ is the propensity score of the k-th treatment, Z is the categorical treatment, and I is the indicator (1 if true, 0 if false). The balance of each covariate across the two comparison groups was checked using standardized differences before and after PS weighting. We made a direct comparison of the median value of the outcome variables in the PS-weighted cohort. Kaplan–Meier survival curves were built from the original and weighted cohorts. The difference in survival curves was tested by a logrank test. Cox proportional hazard models were used to calculate the hazard ratios (HR) and 95% confidence intervals (CI) in both the original cohort (crude HR) and the PS-weighted cohort (PS-weighted HR). To investigate the independent risk factors associated with IHCA, we entered the significant variables on univariate comparison as well as age and gender into a logistic regression model. We used the backward elimination method to select significant independent risk factors. The model was validated using the 10-fold cross-validation. We reported the C-statistics of the original model and optimism-corrected c-statistics to adjust for the overfitting problem. To assess the presence of effect modification, we conducted several pre-specified subgroup analyses of particular clinical importance, including age > or ≤ 75 years, gender, and presence/absence of cancer, cardiovascular disease, diabetes mellitus, acute respiratory failure, lower respiratory tract infection, and genitourinary tract infection. All *p*-values are two-sided, and a *P* < 0.05 was considered statistically significant unless otherwise stated. Data management and statistical analyses were conducted using SAS (version 9.4, SAS Inc., Cary, NC).

## Results

### Study Population Characteristics

**Figure 1:**
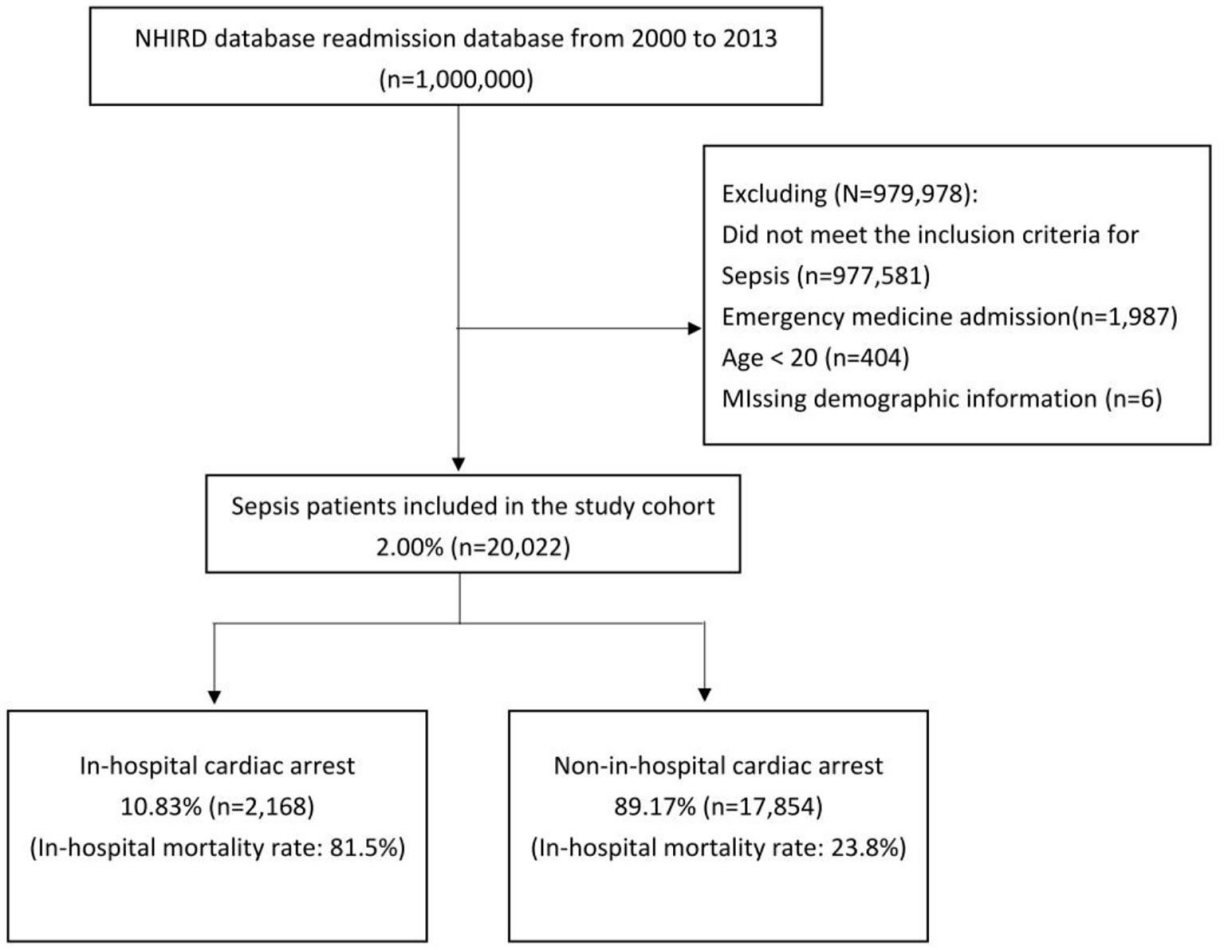
Study cohort. We identified 20,022 patients with sepsis from 2000 to 2013. In this cohort, 10.83% of the patients developed IHCA.

We identified 20,022 patients who met Martin's coding criteria for sepsis from 2000 to 2013, among whom 2,168 (10.83%) patients developed IHCA and received cardiopulmonary resuscitation (Figure 1). The in-hospital mortality rate for patients who developed IHCA was 81.46% compared to 23.83% among patients who did not develop IHCA. Men were more likely to develop IHCA compared to women. Genitourinary tract infection and primary bacteremia were more common among the patients without IHCA compared with those with IHCA, whereas lower respiratory tract infections were common among patients who developed IHCA (Table 1). There were also no statistically significant differences in mean age, urbanization level, the prevalence of coronary artery disease (CAD), diabetes mellitus, chronic pulmonary diseases, malignancy, metastatic disease, and congestive heart failure (CHF) between patients with and without IHCA.

**Table 1:** Comparison of patient characteristics between patients with and without post-sepsis cardiovascular complications at the end of 180 days of follow-up.

**Characteristics**	**Patients with IHCA**	**Patients without IHCA**	***P*-value**
	***N* = 2,168**	***N* = 17,854**	
**Demographics**			
Male sex, *n* (%)	1,450 (66.9%)	10,507 (58.8%)	<0.001^*^
Age, yrs, ± SD	70.82 ± 15.73	70.96 ± 15.61	0.68
**Urbanization level**, ***n*** **(%)**			
Level 1: Urban Area	921 (42.5%)	7,059 (39.5%)	0.001^*^
Level 2: Metro Area	560 (25.8%)	4,323 (24.2%)	
Level 3: Suburban Area	364 (16.8%)	3,385 (19.0%)	
Level 4: Countryside Area	323 (14.9%)	3,087 (17.3%)	
**Insurance premium level, New Taiwan Dollars (%)**			
Dependent	303 (14.0%)	2,498 (14.0%)	<0.001^*^
No/poverty income level ($1–$19,999)	120 (5.5%)	1,485 (8.3%)	
Middle income level ($20,000–$39,999)	1,331 (61.4%)	7,973 (44.7%)	
High income level (≥$40,000)	414 (19.1%)	5,898 (33.0%)	
**Preadmission comorbidity**, ***n*** **(%)**			
Myocardial infarction	44 (2.0%)	402 (2.3%)	0.559
Congestive heart failure	368 (17.0%)	2,752 (15.4%)	0.063
Peripheral vascular disease	80(3.7%)	712 (4.0%)	0.539
Cerebrovascular disease	680 (31.4%)	5,836 (32.7%)	0.224
Dementia	327 (15.1%)	2,776 (15.5%)	0.593
COPD or asthma (496, 49,121,49,320,493.21,493.22)	591(27.3%)	4,187(23.5%)	<0.001^*^
Other chronic pulmonary disease (011.93)	17(0.8%)	108(0.6%)	0.32
Rheumatologic disease	24 (1.1%)	227 (1.3%)	0.584
Peptic ulcer disease	548 (25.3%)	4,582 (25.7%)	0.716
Mild liver disease	319 (14.7%)	2,717 (15.2%)	0.558
Diabetes without chronic complications	672 (31.0%)	5,549 (31.1%)	0.956
Diabetes with chronic complications	251 (11.6%)	1,766 (9.9%)	0.015^*^
Hemiplegia or paraplegia	61(2.8%)	543 (3.0%)	0.604
Renal disease (2,504, 5,849)	366 (16.9%)	2,762 (15.5%)	0.093
Any malignancy, including leukemia and lymphoma	321 (14.8%)	3,085(17.3%)	0.004^*^
Hepatocellular carcinoma or liver cirrhosis (5,715, 5,712, 5,716, 5,722, 155.0–155.2)	182 (8.39%)	1,607(9.00%)	0.35
Other moderate or severe liver disease	53 (2.44%)	434(2.43%)	0.97
Metastatic solid tumor	30 (1.4%)	334 (1.9%)	0.129
AIDS/HIV	4 (0.2%)	26 (0.1)	0.882
Alcohol/drug use	37 (1.7%)	365 (2.0%)	0.328
Psychiatric disorder (295,296)	342 (15.8%)	2,751 (15.4%)	0.678
Neurologic disorder	235(10.8%)	2,113 (11.8%)	0.185
Obesity	4(0.2%)	41 (0.2%)	0.858
Bed-ridden status (7,070)	63 (2.9%)	557 (3.1%)	0.633
Solid organ transplantation	1 (0.0%)	34 (0.2%)	0.213
Atrial fibrillation	86(4.0%)	757 (4.2%)	0.588
Femoral fracture within 1 year (82,021, 8,208)	76(3.5%)	532(3.0%)	0.18
Deep vein thrombosis or pulmonary embolism (44,422, 453.40)	40(1.9%)	350(2.0%)	0.71
**Acute organ dysfunction**, ***n*** **(%)**			
Renal dysfunction	278 (12.8%)	3,101 (17.4%)	<0.001^*^
Cardiovascular dysfunction/Shock	963 (44.4%)	8,184 (45.8%)	0.22
Hematologic system dysfunction	68 (3.1%)	711 (4.0%)	0.06
Acute respiratory failure	1,590 (73.3%)	8,597 (48.2%)	<0.001^*^
CNS dysfunction	50 (2.3%)	388 (2.2%)	0.75
Hepatic dysfunction	10 (0.5%)	252 (1.4%)	<0.001^*^
Gastrointestinal bleeding	137 (6.32%)	811(4.54%)	0.0002^*^
**Source of infection**, ***n*** **(%)**			
Lower respiratory tract infection	862 (39.8%)	6,610 (37.0%)	0.01^*^
Genitourinary tract infection	301 (13.9%)	4,399 (24.6%)	<0.001^*^
Skin and skin structure infection	24 (1.1%)	303 (1.7%)	0.05
Catheter related bloodstream infection	18 (0.8%)	236 (1.3%)	0.07
Intra-abdominal infection	80 (3.7%)	796 (4.5%)	0.11
Biliary tract infection	17 (0.8%)	114 (0.6%)	0.51
Systemic fungal infection	26 (1.2%)	259 (1.5%)	0.40
Primary bacteremia	53 (2.4%)	904 (5.1%)	<0.001^*^
Musculoskeletal infection	11 (0.5%)	134 (0.8%)	0.26

* *Means p-value < 0.05*.

### Risk Factors for IHCA Among Sepsis Patients

**Figure 2:**
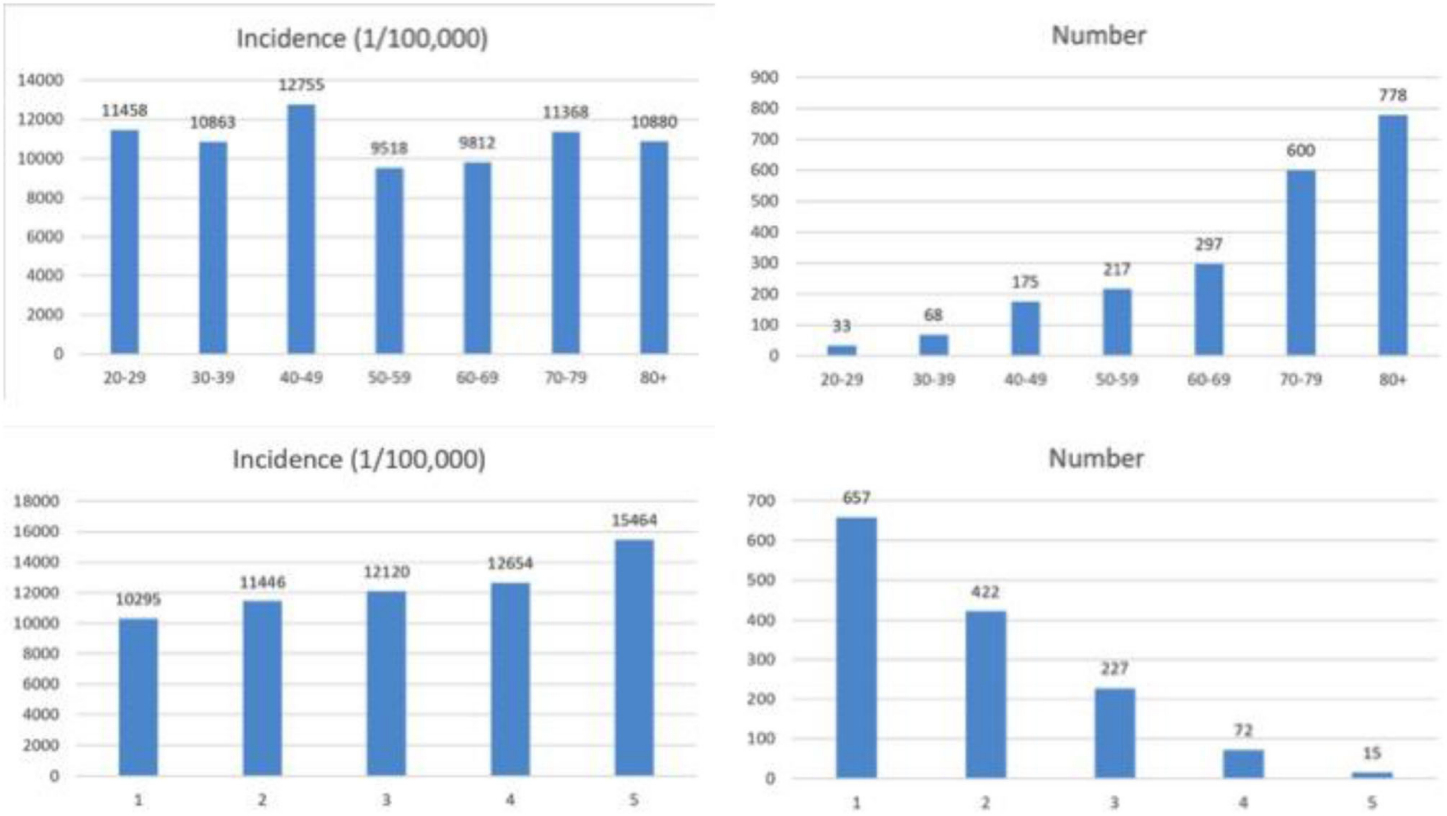
The number of sepsis patients who developed IHCA stratified by age categories or RCRI score.

We hypothesized that the occurrence of IHCA is correlated with age and cardiac risk. Figure 2 shows the number of sepsis patients who developed IHCA stratified by age categories or RCRI score. In our study cohort, IHCA incidence increased with advancing 10-year age categories (*r*^2^ = 0.97, *P* < 0.0001). The incidence of IHCA was also well-calibrated with the RCRI scores. The IHCA risk increased by 1,155 (1/100,000) for each RCRI score increase (*r*^2^ = 0.99, *P* = 0.0006). To evaluate the independent predictors associated with IHCA, we entered the significant variables on univariate comparison (Table 1), as well as age in the 10-year category and revised cardiac index score, into the logistic regression model. We found that acute respiratory failure was the strongest predictor of IHCA with an OR of 2.96 (95% CI: 2.67–3.29), followed by male gender (OR 1.32), psychiatric disorder (OR 1.28), gastrointestinal bleeding (OR 1.26), and RCRI (OR 1.07). The C-statistic for the model, which included all of these covariates, was 0.71 (Table 2).

**Table 2:** Independent risk factors associated with in-hospital cardiac arrest (IHCA) among patients hospitalized for sepsis.

**Risk factor**	**Odds ratio (95% confidence interval)**
Acute respiratory failure	2.96 (2.67, 3.29)
Male gender	1.32 (1.03, 1.12)
Psychiatric disorder	1.28 (1.07, 1.52)
Gastrointestinal bleeding	1.26 (1.04, 1.53)
Revised Cardiac Risk Index	1.07 (1.03, 1.12)
Model statistics	
c-statistics	0.71
Optimism-corrected c-statistics (10-fold CV)	0.70

*Variables were selected using a competing risk model*.

### Impact of IHCA on Survival

**Figure 3:**
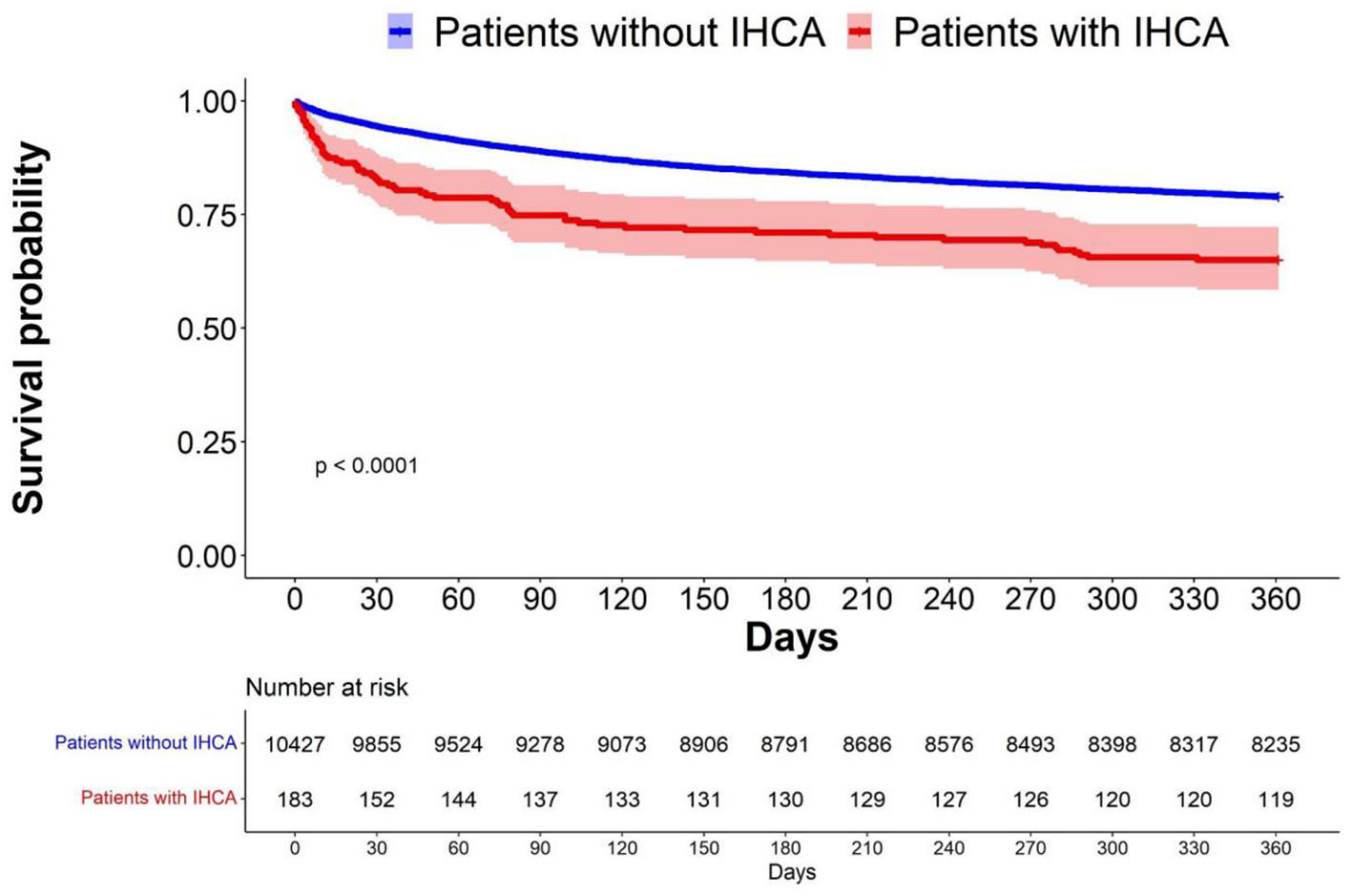
Survival probability before PS weighting.The Kaplan-Meier survival curve showed the survival probability with and without IHCA before PS weighting.

**Figure 4:**
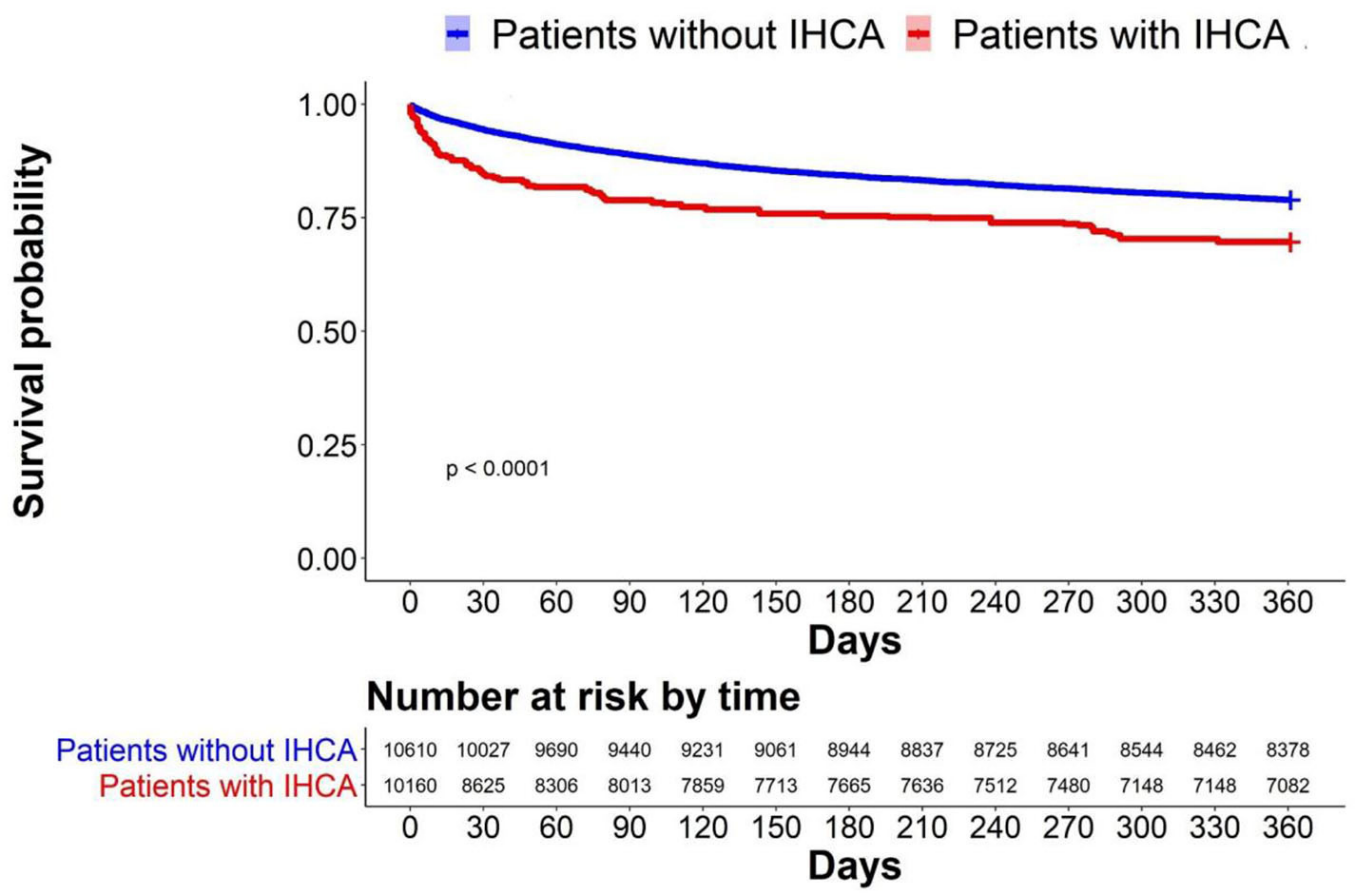
Survival probability after PS weighting.The Kaplan-Meier survival curve showed the impact of IHCA on survival probability.

We plotted the Kaplan–Meier survival curve of patients with IHCA vs. that of patients without IHCA in the original cohort (Figure 3). Patients who developed IHCA had worse survival in the following year, with an early divergence of the KM curves that was maintained throughout the duration of follow-up (Log-rank test, *p* < 0.0001). We evaluated all the factors that might affect the survival of septic patients who experienced IHCA and selected significant covariates through a binary logistic model. The standardized mean difference graph showed that all the selected covariates were balanced in a satisfactory range (<10%) and the PS weighting successfully eliminated the difference in baseline covariates (Supplementary Material). After PS weighting, there was still a significant difference between the survival curve of the patients with IHCA and that of the patients without IHCA (Figure 4, log-rank test, *p* < 0.0001). Sepsis patients with IHCA in the original cohort had a higher 30-day mortality rate (HR, 3.45; 95% CI, 2.37–5.01) and 1 year mortality rate (HR, 1.40; 95% CI, 1.01–1.96) when compared to sepsis patients without IHCA. In the PS-weighted cohort, IHCA patients still had a worse 30-day mortality rate (HR, 3.06; 95% CI, 2.77–3.37) and 1 year mortality rate (HR, 1.07; 95% CI, 1.00–1.15) (Table 3).

**Table 3:** Comparison of post-discharge 30-day or 1 year survival between patients with and without IHCA during hospitalization.

	**Overall**	**IHCA patients**	**Non-IHCA patients**	**HR (95% CI)**	***p*-value**
**Original cohort**					
30-day mortality rate	5.10%	29 (15.85%)	512 (4.91%)	3.45 (2.37, 5.01)	<0.0001
1-year mortality rate	17.04%	35 (22.73%)	1,681 (16.95%)	1.40 (1.01, 1.96)	0.05
**PS weighted cohort**					
30-day mortality rate	9.77%	1,606 (14.37%)	521 (4.91%)	3.06 (2.77, 3.37)	<0.0001
1-year mortality rate	17.52%	1,732 (18.10%)	1,712 (16.97%)	1.07 (1.00, 1.15)	0.04

*Survival differences were calculated as hazard ratio (HR) in the full cohort. We used the PS weighting to adjust for the potential confounding*.

### Subgroup Analysis

We additionally assessed for potential effect modification between baseline characteristics and the impact on survival after IHCA. Results showed that IHCA events had a more pronounced adverse impact on patients with healthier conditions. Patients in the age group of ≤ 75 years or with genitourinary tract infection, or patients without cancer, cardiovascular disease, diabetes mellitus, acute renal failure, or lower respiratory tract infection were more adversely affected by the IHCA events on the 30-day survival than those with these conditions. The interaction term *p*-value was significant at <0.05 (Table 4).

**Table 4:** Subgroup analysis for post-discharge 30-day mortality among sepsis survivors.

	**Post-discharge 30-day mortality**		**HR (95% CI)**	**Interaction P**
	**Patients with IHCA**	**Patients without IHCA**		
Age > 75 years	12.50%	5.81%	2.34 (2.08,2.65)	<0.0001
Age ≤ 75 years	22.22%	3.19%	9.96 (8.14,12.18)	
Male	15.65%	5.62%	3.45 (3.04,3.92)	0.06
Female	16.18%	3.98%	4.25 (3.58,5.03)	
Cancer	11.11%	6.59%	1.26 (1.02,1.55)	<0.0001
Non-cancer	16.67%	4.38%	5.09 (4.51,5.74)	
Cardiovascular disease^*^	14.29%	5.15%	3.31 (2.92,3.75)	0.0006
Non-CV disease	18.75%	4.49%	4.83 (4.05,5.76)	
DM (with complications)	9.80%	4.61%	1.69 (1.31,2.18)	<0.0001
Non-DM	18.18%	4.99%	4.28 (3.82,4.79)	
Acute respiratory failure	12.95%	5.92%	2.40 (2.07,2.78)	<0.0001
Non-ARF	25.00%	4.34%	8.31 (7.17,9.63)	
Lower respiratory tract infection (LRTI)	12.50%	6.47%	2.16 (1.82,2.56)	<0.0001
Non-LRTI	17.65%	4.19%	4.90 (4.30,5.57)	
Genitourinary tract infection	20.00%	3.65%	5.22 (4.19,6.50)	0.0004
Non-GUTI	15.03%	5.44%	3.35 (2.98,3.76)	

* *Cardiovascular disease includes myocardial infarction, cerebrovascular disease, peripheral vascular disease, and congestive heart failure*.

## Discussion

This study described the basic characteristics among sepsis patients with and without IHCA. Five variables were identified as being associated with post-sepsis IHCA in the multivariable Cox regression analysis; these were a respiratory failure, male gender, diabetes with complications, 10-year increase in age, and RCRI. In the survival analysis, patients with post-sepsis IHCA had worse long-term survival compared to patients without IHCA, who had otherwise similar comorbidities. We showed that the increased risk of IHCA was a result of sepsis rather than the general disease condition of the patients. In subgroup analyses, identifying that post-sepsis IHCA affected patients with fewer comorbidities stresses the importance of preventing sepsis IHCA to reduce loss of disability-adjusted life-years (DALYs).

Sepsis-related systemic inflammation exposes patients to higher concentrations of proinflammatory and prothrombotic factors and may be associated with more cellular metabolic dysregulation. This milieu has been associated with an increased risk for cardiovascular events, including IHCA (12). This phenomenon has been supported in preclinical models (13–15) and observed in short- and long-term prospective studies of pneumonia and urinary tract infections (16). This hypothesis is compatible with previous studies in which sepsis-related IHCA portended a worse prognosis than non-sepsis-related IHCA (6). There have been no prior studies that have identified risk factors for IHCA among sepsis patients, making our study the first of its kind to identify novel risk factors.

We demonstrated that IHCA led to worse long-term survival among sepsis patients. From a physiological standpoint, we posit that the patients experiencing IHCA have a higher baseline cardiovascular risk or experience more severe sepsis. IHCA events themselves expose patients to additional degrees of acute injury through systemic hypoxia, poor perfusion, and potentially proarrhythmic medications. They lead to protracted clinical courses and increased number of invasive procedures, alongside exposure to hand-offs and intubation (17). Ultimately, these may result in unavoidable loss of life or withdrawal of care. We suspect that this risk is additive and unequally distributed amongst the sepsis population, given the clear differences in outcomes within our study.

There have been several scoring systems using demographic characteristics, laboratory test results, and physiological parameters to predict the onset of IHCA during hospitalization, such as MEWS, NEWS, and eCART. Bartkowiak et al. (18) showed that for post-operative surgical patients, all those three scoring systems showed good performance in predicting adverse events such as ICD transfer, cardiac arrest, and death. In their study, the area under the ROC curve (AUC) for eCART was 0.79, which showed the best performance among all three scores. In the study of Churpek et al. (19), the results also showed that the MEWS score of 48 h proceeding cardiac arrest had the best performance of predicting cardiac arrest (area under the ROC curve = 0.77). Nevertheless, though those studies suggested that the proximate causes could be more relevant to the onset of IHCA, more accurate algorithms used to predict IHCA for sepsis patients based on baseline comorbidities identified at admission are still needed by physicians. Such algorithms will help physicians to identify high-risk sepsis patients and to better allocate resources. Our results showed that for sepsis patients, using an algorithm based on baseline comorbidities can predict the onset of IHCA. Though the differences among study populations may affect the AUROC, our study using nationwide claims data showed an AUROC of 0.70 after 10-fold validation, which provides plausible generalizability.

In our algorithm, we used the RCRI as a surrogate measure of cardiovascular disease burden in order to identify patients who were more likely to undergo these cardiovascular events. This is the first demonstration of using RCRI to identify the risk of IHCA in sepsis patients and warrants more research. Within the perioperative evaluation, RCRI was found to be inferior to National Surgical Quality Improvement Program (NS-QIP) when predicting the incidence of cardiac arrest (20). However, in this application, RCRI can be advantageous as it uses fewer variables to calculate risk and can serve as a simple tool to identify high-risk sepsis patients. We recommend future investigation for a better predictive metric that incorporates the use of RCRI to characterize cardiovascular risk in addition to the risk factors identified by us.

The strengths of this study include the large, nationally representative data with over 20,000 sepsis patients, and the identified novel risk factors which were associated with long-term adverse impact on IHCA. However, there are a few important limitations within our study. Our data spans hospitalizations during the period of 2000–2013 and demonstrates a very high rate of IHCA. We believe this is the result of including patients with sepsis and by including hospitalizations before widespread adoption of rapid-response teams, code teams, and training on best practices in CPR. We cannot meaningfully speculate that our event rates would be similar in 2021, but would expect risk factors to be similar as stated previously. Since our analysis employed a database, we did not have information regarding rhythm data, timing, medications, and laboratory studies associated with IHCA resuscitation episodes. Thus, we were unable to evaluate the impact of these factors on long-term outcomes. Further areas of study should include factors associated with post-discharge survival among sepsis patients following IHCA. This would provide insight into improving long-term outcomes in these patients.

## Conclusion

In-hospital cardiac arrest is a complication of sepsis and its incidence rises with increasing age and pre-existing cardiac risk. Furthermore, the adverse effects of IHCA extend beyond the hospitalization course to 1 year after discharge. The three newly identified risk factors or RCRI scores can be used to identify patients who are at high risk for developing IHCA. As IHCA has been shown to be preventable, our study should prompt further studies that aim to include IHCA prevention as part of sepsis care quality improvement activity.

## Data Availability Statement

Publicly available datasets were analyzed in this study. This data can be found at: National Health Insurance Research Database of Taiwan (NHRID).

## Ethics Statement

This study was approved by the Institutional Review Board at National Taiwan University Hospital, which waived the requirement for informed consent from patients because of the anonymous nature of the data.

## Author Contributions

C-CL had full access to all the data in the study, took responsibility for the integrity of the data, the accuracy of the data analysis, concept and design, critical revision of the manuscript for important intellectual content, obtaining funding, and supervision. CD, CS, FQ, J-CH, and D-AL drafted the manuscript, were responsible for the interpretation of the data, and critical revision of the manuscript for important intellectual content. I-JW, YL, CW, and BT were responsible for the critical revision of the manuscript for important intellectual content. W-TH was responsible for the concept and design and critical revision of the manuscript for important intellectual content. T-CH was responsible for statistical analysis and critical revision of the manuscript for important intellectual content. All authors read and approved the final manuscript.

## Funding

This study was supported by MOST 105-2811-B-002-031.

## Conflict of Interest

The authors declare that the research was conducted in the absence of any commercial or financial relationships that could be construed as a potential conflict of interest.

## Publisher's Note

All claims expressed in this article are solely those of the authors and do not necessarily represent those of their affiliated organizations, or those of the publisher, the editors and the reviewers. Any product that may be evaluated in this article, or claim that may be made by its manufacturer, is not guaranteed or endorsed by the publisher.

## Supplementary Material

Supplementary Material The standardized mean difference graph showed that the covariates were balanced in a satisfactory range (< 10%). The PS weighting was successful.

## References

[1] Andersen LW Holmberg MJ Berg KM Donnino MW Granfeldt A. In-hospital cardiac arrest: a review. J Am Med Assoc. (2019) 321:1200–10. 10.1001/jama.2019.1696 30912843 PMC6482460

[2] Chen LM Nallamothu BK Spertus JA Li Y Chan PS. Association between a hospital's rate of cardiac arrest incidence and cardiac arrest survival. J Am Med Assoc Intern Med. (2013) 173:1186–95. 10.1001/jamainternmed.2013.1026 23689900 PMC4181325

[3] Layeghian Javan S Sepehri MM Layeghian Javan M Khatibi T. An intelligent warning model for early prediction of cardiac arrest in sepsis patients. Comput Methods Programs Biomed. (2019) 178:47–58. 10.1016/j.cmpb.2019.06.010 31416562

[4] Perman SM Stanton E Soar J Berg RA Donnino MW Mikkelsen ME. Location of in-hospital cardiac arrest in the United States-variability in event rate and outcomes. J Am Heart Assoc. (2016) 5:3638. 10.1161/JAHA.116.003638 27688235 PMC5121474

[5] Larkin GL Copes WS Nathanson BH Kaye W. Pre-resuscitation factors associated with mortality in 49,130 cases of in-hospital cardiac arrest: a report from the National Registry for Cardiopulmonary Resuscitation. Resuscitation. (2010) 81:302–11. 10.1016/j.resuscitation.2009.11.021 20047786

[6] Koivikko P Arola O Inkinen O Tallgren M. One-year survival after inhospital cardiac arrest-does prearrest sepsis matter? Shock. (2018) 50:38–43. 10.1097/SHK.0000000000001024 29889807

[7] Singer M Deutschman CS Seymour CW Shanker-Hari M Annane D Bauer M. The third international consensus definitions for sepsis and septic shock (Sepsis-3). J Am Med Assoc. (2016) 315:801–10. 10.1001/jama.2016.0287 26903338 PMC4968574

[8] Martin GS Mannino DM Eaton S Moss M. The Epidemiology of Sepsis in the United States From 1979 Through 2000. N Engl. J. Med. (2003) 348:1546–54. 10.1056/NEJMoa022139 12700374

[9] Rhee C Dantes R Epstein L Murphy DJ Seymour CW Iwashyna J. Incidence and trends of sepsis in US hospitals using clinical vs claims data, 2009–2014. J Am Med Assoc. (2017) 318:1241. 10.1001/jama.2017.13836 28903154 PMC5710396

[10] Fleisher LA Beckman JA Brown KA Calkins H Chaikof KL Fleishmann KE. 2009 ACCF/AHA focused update on perioperative beta blockade incorporated into the ACC/AHA 2007 guidelines on perioperative cardiovascular evaluation and care for non-cardiac surgery: a report of the American college of cardiology foundation/American heart association task force on practice guidelines. Circulation. (2009) 120:e169–276. 10.1161/CIRCULATIONAHA.109.192690 19884473

[11] Yoshida K Hernández-Díaz S Solomon DH Jackson JW Gagne JJ Glynn RJ. Matching weights to simultaneously compare three treatment groups: comparison to three-way matching. Epidemiology. (2017) 28:387–95. 10.1097/EDE.0000000000000627 28151746 PMC5378668

[12] Ramirez J Aliberti S Mirsaeidi M Peyrani P Filardo G Amir A. Acute myocardial infarction in hospitalized patients with community-acquired pneumonia. Clin Infect Dis. (2008) 47:182–7. 10.1086/589246 18533841

[13] Fenton KE Parker MM. Cardiac function and dysfunction in sepsis. Clin Chest Med. (2016) 37:289–98. 10.1016/j.ccm.2016.01.014 27229645

[14] Merx MW Weber C. Sepsis and the heart. Circulation. (2007) 116:793–802. 10.1161/CIRCULATIONAHA.106.678359 17698745

[15] Bosmann M Ward PA. The inflammatory response in sepsis. Trends Immunol. (2013) 34:129–36. 10.1016/j.it.2012.09.004 23036432 PMC3543471

[16] Musher DM Abers MS Corrales-Medina VF. Acute infection and myocardial infarction. N Engl J Med. (2019) 380:171–6. 10.1056/NEJMra1808137 30625066

[17] Graham R McCoy MA Schultz AM. Directions C on the T of CACS and F, Policy B on HS, Medicine I of. In-Hospital Cardiac Arrest and Post-Arrest Care. National Academies Press (US) (2015). Available online at: https://www.ncbi.nlm.nih.gov/books/NBK321499/ (accessed February 1, 2021).

[18] Bartkowiak B Snyder AM Benjamin A Schneider A Twu NM Churpek MM. Validating the electronic cardiac arrest risk triage (eCART) score for risk stratification of surgical inpatients in the postoperative setting: retrospective cohort study. Ann Surg. (2019) 269:1059–63. 10.1097/SLA.0000000000002665 31082902 PMC6610875

[19] Churpek MM Yuen TC Huber MT Park SY Hall JB Edelson DP. Predicting cardiac arrest on the wards: a nested case-control study. Chest. (2012) 141:1170–6. 10.1378/chest.11-1301 22052772 PMC3342781

[20] Guarracino F Baldassarri R Priebe HJ. Revised ESC/ESA Guidelines on non-cardiac surgery: cardiovascular assessment and management. Implications for preoperative clinical evaluation. Minerva Anestesiol. (2015) 81:226–33. 25384693

