# Supplementary figures and images for: In-hospital Cardiac Arrest in Patients With Sepsis: A National Cohort Study
Source: Front Med (Lausanne). 2021 Oct 15;8:731266. doi: 10.3389/fmed.2021.731266 (PMC8553946; doi:10.3389/fmed.2021.731266)

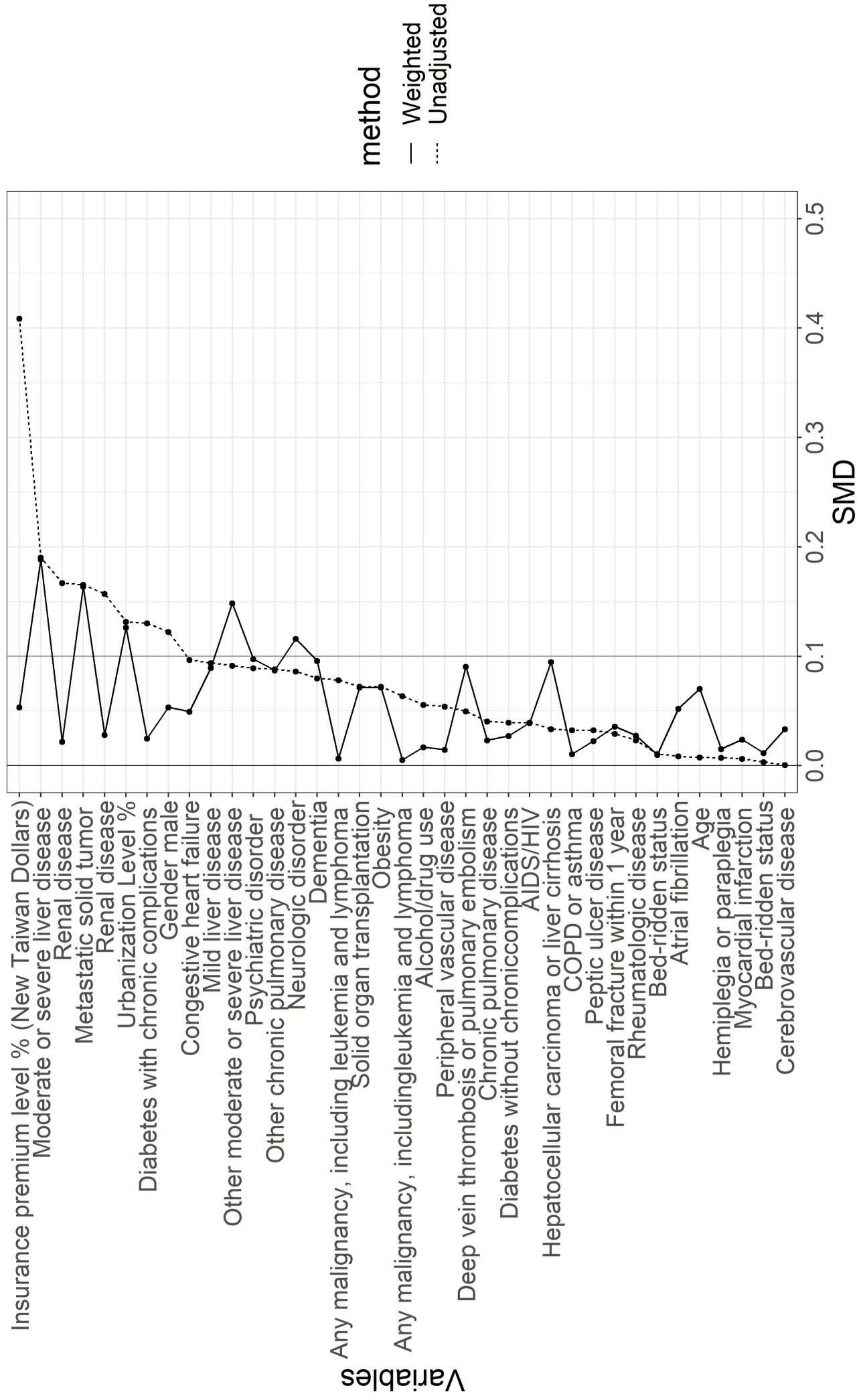

Supplement: Supplementary Material — The standardized mean difference graph showed that the covariates were balanced in a satisfactory range (< 10%). The PS weighting was successful. [file Data_Sheet_1.PDF]
